# Comparison of prescribing practices for older adults treated by female versus male physicians: A retrospective cohort study

**DOI:** 10.1371/journal.pone.0205524

**Published:** 2018-10-22

**Authors:** Paula A. Rochon, Andrea Gruneir, Chaim M. Bell, Rachel Savage, Sudeep S. Gill, Wei Wu, Vasily Giannakeas, Nathan M. Stall, Dallas P. Seitz, Sharon-Lise Normand, Lynn Zhu, Nathan Herrmann, Lisa McCarthy, Colin Faulkner, Jerry H. Gurwitz, Peter C. Austin, Susan E. Bronskill

**Affiliations:** 1 Women’s College Research Institute, Women’s College Hospital, Toronto, Ontario, Canada; 2 Department of Medicine, University of Toronto, Toronto, Ontario, Canada; 3 Institute of Health Policy, Management, and Evaluation, University of Toronto, Toronto, Ontario, Canada; 4 Institute for Clinical Evaluative Sciences, Toronto, Ontario, Canada; 5 Department of Family Medicine, University of Alberta, Edmonton, Alberta, Canada; 6 Sinai Health System, Toronto, Ontario, Canada; 7 Department of Medicine, Queen’s University, Kingston, Ontario, Canada; 8 Institute for Clinical Evaluative Sciences, Kingston, Ontario, Canada; 9 Division of Geriatric Psychiatry, Department of Psychiatry, Queen’s University, Kingston, Ontario, Canada; 10 Department of Health Care Policy, Harvard Medical School, Boston, Massachusetts, United States of America; 11 Department of Biostatistics, Harvard T.H. Chan School of Public Health, Boston, Massachusetts, United States of America; 12 Department of Psychiatry, University of Toronto, Toronto, Ontario, Canada; 13 Leslie Dan Faculty of Pharmacy, University of Toronto, Toronto, Ontario, Canada; 14 Division of Geriatric Medicine, Department of Medicine, University of Massachusetts Medical School, Worcester, Massachusetts, United States of America; University of Sydney, AUSTRALIA

## Abstract

**Importance:**

Subtle but important differences have been described in the way that male and female physicians care for their patients, with some evidence suggesting women are more likely to adhere to best practice recommendations.

**Objective:**

To determine if male and female physicians differ in their prescribing practices as measured by the initiation of lower-than-recommended dose cholinesterase inhibitor (ChEI) drug therapy for dementia management.

**Design, setting, and participants:**

All community-dwelling Ontario residents aged 66 years and older with dementia and newly dispensed an oral ChEI drug (donepezil, galantamine, or rivastigmine) between April 1, 2010 and June 30, 2016 were included.

**Main outcome and measures:**

The association between physician sex and the initiation of a lower than recommended-dose ChEI was examined using generalized linear mixed regression models, adjusting for patient and physician characteristics. Data were stratified by specialty. Secondary analyses explored the association between physician sex and cardiac screening as well as shorter duration of the initial prescription.

**Results:**

The analysis included 3,443 female and 5,811 male physicians and the majority (83%) were family physicians, Female physicians were more likely to initiate ChEI therapy at a lower-than-recommended dose (Adjusted odds ratio = 1.43,95% confidence interval = 1.17 to 1.74). Compared to their male counterparts, female physicians were also more likely to follow other conservative prescribing practices including cardiac screening (55.1% vs. 49.2%, P-value<0.001) around the time of ChEI initiation, and dispensing a shorter duration of initial prescription (41.8% vs 35.5% P-value<0.001).

**Conclusions:**

There is a statistically significant and important difference in ChEI prescribing patterns between female and male physicians, suggesting that female physicians may be more careful and conservative in their approaches. This will inform future research to determine if patients receiving lower-than-recommended initial doses also have better outcomes.

## Introduction

A growing body of evidence supports differences in the style of medicine practiced by physicians according to their sex and gender. Relative to male physicians, evidence suggests that female physicians spend more time with their patients[1], provide more counselling about unhealthy behaviours[2], are more likely to adhere to guidelines[3] and deliver recommended screening[4, 5], and offer more follow-up care[1]. Taken collectively, these differences suggest that female physicians pursue a more careful and conservative approach to patient management. There is also some evidence that this approach may result in female physicians providing better outcomes including: enhanced management of patients with certain chronic conditions[6], fewer emergency room visits or hospitalizations[7], lower hospital readmission and mortality rates[8], and better postoperative outcomes[9].

In the context of drug prescribing, the most frequent physician intervention, differences in the process of drug prescribing between male and female physicians has not been well described. Initiating a drug therapy at a low-dose is one measure of a more careful and conservative prescribing practice because adverse events are often dose-related [10]. Careful is defined as “applying care, solicitous attention, or pains to what one has to do; heedful, painstaking, attentive to one’s work”[11]. Conservative is defined as “conserves, or favours the conservation of, an existing structure or system; designating a person, movement, outlook, etc., averse to change or innovation and holding traditional ideas and values” [12]. Prescribing low-dose therapy is a well-documented practice used to minimize dose-related adverse events while providing benefits [10]. For some frequently used drug therapies, this dose is lower than the lowest manufactured dose and requires pill-splitting [10]. Conventional wisdom in geriatric medicine is “start low and go slow”. Where possible drug therapy should be initiated in an older individual at a low-dose[13].

Cholinesterase inhibitor (ChEI) drug therapy provides an excellent model to explore subtle differences in the way that female and male physicians prescribe low-dose drug therapy. First, ChEI therapy is widely used internationally because it is one of the only drugs available for the management of dementia. Second, ChEI therapy provides only modest benefit on cognition, and some evidence challenges the scientific basis for recommending this therapy[14]. Finally, this therapy is associated with dose-related adverse events [15]. As such there is little urgency to achieve a maximum therapeutic dose quickly, making it important to consider a more careful and conservative approach to drug initiation to promote patient safety [16]. This model provides the opportunity to learn about the small number of physicians who prescribe a lower-than recommended staring dose of a ChEI therapy suggesting that they are following a more careful and conservative approach to their prescribing.

To explore more careful and conservative prescribing practices between female and male physicians, we examined incident use of ChEI drug therapy in a population-based cohort of older adults with dementia. Analyses were conducted separately by physician speciality.

## Materials and methods

### Data sources

We conducted a population-based retrospective cohort study using administrative healthcare data from Ontario, Canada. Ontario has a population of close to 1.8 million adults aged 65 and older who have universal health and drug coverage. These data have been used for a range of studies exploring drug therapy in older adults.[17–19] All older adults receive universal health coverage that includes most physician services, hospital admissions, and outpatient prescription drugs available through the Ontario Drug Benefit (ODB) Plan. This study used nine administrative healthcare databases that were linked using unique encoded identifiers and analyzed at the Institute for Clinical Evaluative Sciences (ICES). All data in the study were fully anonymized. The information about the patient and physician records is shown in S1 File.

This study was approved by the Research Ethics Board of Sunnybrook Health Sciences Centre.

### Cohort definition

All community-dwelling Ontario residents aged 66 years and older with dementia and newly dispensed an oral ChEI drug (donepezil, galantamine, or rivastigmine) between April 1, 2010 and June 30, 2016 were included. The N-methyl-D-aspartate receptor antagonist memantine was not included because it is not reimbursed through the ODB. A lower age limit of 66 years was used to allow for at least one year of enrolment in the ODB program. The index date was the first dispensed ChEI in the time window. New users were defined as those who had not received another ChEI prescription in the year prior to index. To be considered community-dwelling, individuals could not reside in a nursing home in the year prior to initiating ChEI therapy. Nursing home residents were excluded because they differ from older adults in the community in several ways including their frailty, comorbidity, as well as the lower likelihood of newly initiating a ChEI. A validated data algorithm[20] based on hospital diagnoses, physician records, and prescription drug claims was used to identify individuals with prevalent dementia[20].

### Exposure: Sex of the prescribing physician

Each patient newly dispensed a ChEI was assigned to a prescribing physician. The prescribing physician was identified using unique encoded physician identifiers in the ODB database. These physician identifiers permit linkage with the ICES Physician Database. We excluded patients whose physician identifier was missing or whose profile did not include their sex.

### Primary outcomes: More careful and conservative prescribing practice

The primary outcome measure was the initiation of low-dose ChEI therapy and defined as a lower-than-recommended starting dose. For ChEI therapy, some resources recommend taking a more careful and conservative approach and starting at lower-than-recommended starting doses[21, 22]. This is because like many drug therapies, ChEI adverse events are dose-related[15]. For example, the donepezil product monograph[23] recommends not increasing the dose for frail older women beyond the recommended starting dose (i.e. 5 mg)[24] to prevent dose-related adverse events.

The initial dose of the ChEI therapy was calculated using information available from ODB including the quantity of the drug dispensed (e.g. number of tablets), the number of days supplied, and the drug identification number (DIN). The DIN signifies information about each drug including strength. For example, if a patient was dispensed a 30-day supply of a 5 mg tablet of donepezil and given 30 tablets, their dose was classified as 5 mg per day.

Each ChEI therapy was assigned to one of three mutually exclusive dose categories: lower-than-recommended starting dose, recommended starting dose, higher-than-recommended starting dose based on dosing recommendations provided in standard drug prescribing textbooks[24], the product monographs[15, 23, 25] and using an approach followed in our prior research[26] (S1 Table).

### Secondary outcomes: Cardiac screening and shorter duration of prescription

Screening for possible cardiac contraindications to ChEI use prior to the index date was considered as secondary outcome and considered as an additional aspect of a more careful and conservative practice. This is because ChEI therapy can cause bradycardia or syncopal episodes[27]. Product monographs for donepezil[23], galantamine[15] and rivastigmine[25] recommend that ChEI should not be used in those with cardiac conduction abnormalities[24]. Our hypothesis was that a more careful and conservative prescriber would screen for these cardiac contraindications prior to initiating ChEI. This measure was operationalized as evidence of cardiac screening (i.e. an assessment by a cardiologist, an electrocardiogram (ECG), or a Holter monitor) obtained in the six months prior to initiating a ChEI therapy. The six-month time period was selected because we believed it represented a time period where the assessments would be considered current.

Dispensing initial prescription for a short duration, which may facilitate monitoring of adverse events, was also considered as a measure of a more careful and conservative practice. The ChEI drug monograph recommends monitoring closely following drug initiation to avoid or decrease adverse events[23]. Our hypothesis was that a more careful and conservative prescriber would give a short duration of initial ChEI. Specifically, we measured the length in days that the initial ChEI prescription was dispensed and less than 30 days was defined as a short duration.

### Covariates

#### Physician characteristics

Physician characteristics included age, years in practice, Canadian versus international medical graduate status, specialty (i.e. family medicine/general practice, dementia specialist (i.e. geriatric medicine, neurology, psychiatry) or non-dementia related specialist, and location of practice (i.e. urban or rural[28]).

#### Patient characteristics

Patient demographic characteristics included their sex, age at index date, low-income status (according to eligibility for a low-income subsidy in the ODB), and urban versus rural residence. Time since the first documentation of dementia was measured as days between when an individual first met the criteria for the dementia algorithm and the index prescription date. Comorbidity at the time of the index prescription was measured using the Johns Hopkins Aggregated Diagnosis Groups (ADGs) system (version 10.0.1) over a two-year look-back period[29].

### Statistical analyses

Within each group, the distribution of all baseline covariates between male and female physicians was examined and a 0.10 cut-off in standardized differences, or a 10% difference, was used to identify an imbalance between groups[30, 31].

To examine the association between physician sex and the initial dose of the incident ChEI, we used a generalized linear mixed regression model. The model was adjusted for patient characteristics. To account for potential correlation between patient outcomes within the same physician, the models incorporated physician-specific random effects. We were selective in the inclusion of physician characteristics in our models to avoid correcting for factors that may reflect the gender differences that we are trying to study. As such, our analyses were stratified based on medical specialty because the characteristics of the physician and their patients differed by specialty.

We took two steps to evaluate whether differences in dose dispensed between female and male physicians could be accounted for by other physician characteristics. First, we stratified our analyses by specialty (family medicine, dementia specialists, or non-dementia specialists) because our preliminary analyses showed differences between speciality groups in their demographic characteristics and prescribing practices. Second, we adjusted for physicians’ number of years practicing medicine.

Chi-square tests were used to assess the association between prescriber sex and each of the secondary outcomes (cardiac screening and shorter duration of initial prescription).

Analyses were performed using SAS statistical software, version 9.4 (SAS Institute, Cary, North Carolina).

## Results

### Demographic characteristics

The cohort included 73,111 community-dwelling older adults with dementia who were 66 years of age and or older and were newly dispensed a ChEI; 43,572 (59.6%) were women and 29,539 (40.4%) were men (Table 1).

**Table 1:** Physician and patient characteristics of older adults with dementia and initiated cholinesterase inhibitor, by physician speciality.

Characteristic	Overall			Family Medicine			Dementia Specialist (Geriatric Medicine, Psychiatry, or Neurology)			Non-Dementia Specialist		
	Female Physician (n = 3,443)	Male Physician (n = 5,811)	SDif	Female Physician (n = 2,979)	Male Physician (n = 4,739)	SDif	Female Physician (n = 253)	Male Physician (n = 486)	SDif	Female Physician (n = 211)	Male Physician (n = 586)	SDif
**Physicians**												
Age in years												
Median(IQR)	49 (41–58)	58 (48–66)	0.67	49 (40–58)	58 (49–67)	0.69	50 (42–58)	58 (47–66)	0.55	47 (40–58)	56 (45–66)	0.57
Years in practice												
Median(IQR)	22 (13–30)	30 (22–40)	0.66	22 (13–30)	30 (22–40)	0.67	22 (14–30)	30 (21–40)	0.63	21 (13–30)	29 (18–40)	0.6
Canadian medical graduate	2,504 (72.7%)	4,139 (71.2%)	0.03	2,198 (73.8%)	3,491 (73.7%)	0	172 (68.0%)	265 (54.5%)	0.28	134 (63.5%)	383 (65.4%)	0.04
Location of practice:												
Missing	44 (1.3%)	54 (0.9%)	0.03	38 (1.3%)	46 (1.0%)	0.03	< = 5 (1.6%)	< = 5 (0.6%)	0.09	< = 5 (0.9%)	< = 5 (0.9%)	0.01
Urban	3,068 (89.1%)	5,116 (88.0%)	0.03	2,616 (87.8%)	4,072 (85.9%)	0.06	247 (97.6%)	477 (98.1%)	0.04	205 (97.2%)	567 (96.8%)	0.02
Rural	331 (9.6%)	641 (11.0%)	0.05	325 (10.9%)	621 (13.1%)	0.07	< = 5 (0.8%)	6 (1.2%)	0.04	< = 5 (1.9%)	14 (2.4%)	0.03
Number of patients initiated cholinesterase inhibitor												
Median(IQR)	3 (1–6)	4 (1–9)	0.25	3 (1–6)	4 (2–9)	0.4	7 (1–31)	4 (1–19)	0.2	1 (1–3)	1 (1–2)	0.22
**Dementia Patients**												
Number of patients	N = 23,139	N = 49,972		N = 13,655	N = 33,945		N = 8,522	N = 14,315		N = 962	N = 1,712	
Sex												
Female	15,176 (65.6%)	28,396 (56.8%)	0.18	9,443 (69.2%)	19,439 (57.3%)	0.25	5,171 (60.7%)	8,003 (55.9%)	0.1	562 (58.4%)	954 (55.7%)	0.05
Age, years												
66–69	1,204 (5.2%)	2,586 (5.2%)	0	605 (4.4%)	1,429 (4.2%)	0.01	565 (6.6%)	1,073 (7.5%)	0.03	34 (3.5%)	84 (4.9%)	0.07
70–74	2,920 (12.6%)	6,113 (12.2%)	0.01	1,561 (11.4%)	3,751 (11.1%)	0.01	1,219 (14.3%)	2,167 (15.1%)	0.02	140 (14.6%)	195 (11.4%)	0.09
75–79	5,156 (22.3%)	10,965 (21.9%)	0.01	2,891 (21.2%)	7,121 (21.0%)	0	2,017 (23.7%)	3,470 (24.2%)	0.01	248 (25.8%)	374 (21.8%)	0.09
80–84	6,654 (28.8%)	14,239 (28.5%)	0.01	3,944 (28.9%)	9,798 (28.9%)	0	2,453 (28.8%)	3,947 (27.6%)	0.03	257 (26.7%)	494 (28.9%)	0.05
85–89	5,207 (22.5%)	11,302 (22.6%)	0	3,256 (23.8%)	8,125 (23.9%)	0	1,741 (20.4%)	2,777 (19.4%)	0.03	210 (21.8%)	400 (23.4%)	0.04
90+	1,998 (8.6%)	4,767 (9.5%)	0.03	1,398 (10.2%)	3,721 (11.0%)	0.02	527 (6.2%)	881 (6.2%)	0	73 (7.6%)	165 (9.6%)	0.07
Low-income senior	5,005 (21.6%)	10,892 (21.8%)	0	2,980 (21.8%)	7,463 (22.0%)	0	1,761 (20.7%)	3,010 (21.0%)	0.01	264 (27.4%)	419 (24.5%)	0.07
Rural residence	2,734 (11.8%)	6,502 (13.0%)	0.04	2,209 (16.2%)	5,344 (15.7%)	0.01	460 (5.4%)	1,051 (7.3%)	0.08	65 (6.8%)	107 (6.3%)	0.02
Time since the first documentation of dementia prior to cohort entry												
0 days (same day as cohort entry)	12,348 (53.4%)	29,914 (59.9%)	0.13	8,020 (58.7%)	22,173 (65.3%)	0.14	3,851 (45.2%)	6,776 (47.3%)	0.04	477 (49.6%)	965 (56.4%)	0.14
1–179 days	5,570 (24.1%)	9,894 (19.8%)	0.1	2,911 (21.3%)	5,605 (16.5%)	0.12	2,392 (28.1%)	3,882 (27.1%)	0.02	267 (27.8%)	407 (23.8%)	0.09
180–364 days	1,481 (6.4%)	2,649 (5.3%)	0.05	697 (5.1%)	1,485 (4.4%)	0.03	725 (8.5%)	1,083 (7.6%)	0.03	59 (6.1%)	81 (4.7%)	0.06
1–2 years	1,368 (5.9%)	2,642 (5.3%)	0.03	683 (5.0%)	1,550 (4.6%)	0.02	624 (7.3%)	1,007 (7.0%)	0.01	61 (6.3%)	85 (5.0%)	0.06
2+ years	2,372 (10.3%)	4,873 (9.8%)	0.02	1,344 (9.8%)	3,132 (9.2%)	0.02	930 (10.9%)	1,567 (10.9%)	0	98 (10.2%)	174 (10.2%)	0
Aggregated diagnosis groups (ADGs)												
0–4	2,593 (11.2%)	6,304 (12.6%)	0.04	1,799 (13.2%)	4,859 (14.3%)	0.03	729 (8.6%)	1,290 (9.0%)	0.02	65 (6.8%)	155 (9.1%)	0.09
5–9	9,949 (43.0%)	22,373 (44.8%)	0.04	6,000 (43.9%)	15,409 (45.4%)	0.03	3,592 (42.1%)	6,304 (44.0%)	0.04	357 (37.1%)	660 (38.6%)	0.03
10+	10,597 (45.8%)	21,295 (42.6%)	0.06	5,856 (42.9%)	13,677 (40.3%)	0.05	4,201 (49.3%)	6,721 (47.0%)	0.05	540 (56.1%)	897 (52.4%)	0.08
Concurrent medications												
Median (IQR)	4 (2–7)	4 (2–7)	0	4 (2–7)	5 (2–7)	0.01	4 (2–7)	4 (2–7)	0.03	5 (3–8)	5 (3–7)	0.05
0 (ChEI only)	1,281 (5.5%)	2,990 (6.0%)	0.02	706 (5.2%)	1,921 (5.7%)	0.02	532 (6.2%)	987 (6.9%)	0.03	43 (4.5%)	82 (4.8%)	0.02
1–4	10,413 (45.0%)	22,279 (44.6%)	0.01	6,164 (45.1%)	14,962 (44.1%)	0.02	3,886 (45.6%)	6,665 (46.6%)	0.02	363 (37.7%)	652 (38.1%)	0.01
5–9	9,320 (40.3%)	20,157 (40.3%)	0	5,511 (40.4%)	13,842 (40.8%)	0.01	3,408 (40.0%)	5,545 (38.7%)	0.03	401 (41.7%)	770 (45.0%)	0.07
10+	2,125 (9.2%)	4,546 (9.1%)	0	1,274 (9.3%)	3,220 (9.5%)	0.01	696 (8.2%)	1,118 (7.8%)	0.01	155 (16.1%)	208 (12.1%)	0.11
ChEI type												
Donepezil	17,870 (77.2%)	38,572 (77.2%)	0	10,948 (80.2%)	26,822 (79.0%)	0.03	6,176 (72.5%)	10,467 (73.1%)	0.01	746 (77.5%)	1,283 (74.9%)	0.06
Galantamine	4,244 (18.3%)	9,653 (19.3%)	0.02	2,285 (16.7%)	6,042 (17.8%)	0.03	1,782 (20.9%)	3,251 (22.7%)	0.04	177 (18.4%)	360 (21.0%)	0.07
Rivastigmine	1,025 (4.4%)	1,747 (3.5%)	0.05	422 (3.1%)	1,081 (3.2%)	0.01	564 (6.6%)	597 (4.2%)	0.11	39 (4.1%)	69 (4.0%)	0

SDif = Standardized difference, IQR = Interquartile range, ChEI = Cholinesterase inhibitor

### Physician characteristics

A total of 9,254 physician prescribers of ChEI therapy were identified (3,443 (37.2%) female and 5,811 (62.8%) male physicians). At the time of the first ChEI prescription for the patients in the cohort, male physicians had been in practice longer (median 30 years) than their female counterparts (median 22 years, standard difference [SDif] = 0.66). Compared to male physicians, females were younger (median age: 49 vs. 58, SDif = 0.67), more likely to be in family medicine (86.5% vs 81.6%, SDif = 0.15), had fewer patients initiated on ChEI (median number: 3 vs. 4, SDif = 0.25) and were more likely to care for female patients (65.6% vs 56.8%, SDif = 0.18).

Use of a lower-than-recommended initial dose of ChEI therapy differed by physician specialty. Among those in family medicine, 4.1% dispensed the initial ChEI therapy at a lower-than-recommended dose, compared to 6.1% of dementia specialists (Table 2). Further, the rate of lower-than-recommended initial dose therapy use differed within dementia specialists, with geriatricians having the highest rate of lower-than-recommended initial dose prescribing (7.7% in geriatric medicine, 6.8% in psychiatry, and 1.5% in neurology).

**Table 2:** Prescribing carefulness measurement by physician specialty.

Characteristic	Overall			Family Medicine			Dementia Specialist (Geriatric Medicine, Psychiatry, or Neurology)			Non-Dementia Specialist		
	Female Physician (n = 3,443)	Male Physician (n = 5,811)	*P*	Female Physician (n = 2,979)	Male Physician (n = 4,739)	*P*	Female Physician (n = 253)	Male Physician (n = 486)	*P*	Female Physician (n = 211)	Male Physician (n = 586)	*P*
**Primary outcome**												
Initial ChEI starting dose												
Lower-than-recommended	1,418 (6.1%)	2,034 (4.1%)	< .001	706 (5.2%)	1,264 (3.7%)	< .001	684 (8.0%)	712 (5.0%)	< .001	28 (2.9%)	58 (3.4%)	< .001
Recommended	20,139 (87.0%)	44,130 (88.3%)		11,727 (85.9%)	29,602 (87.2%)		7,531 (88.4%)	12,992 (90.8%)		881 (91.6%)	1,536 (89.7%)	
Higher-than-recommended	1,582 (6.8%)	3,808 (7.6%)		1,222 (8.9%)	3,079 (9.1%)		307 (3.6%)	611 (4.3%)		53 (5.5%)	118 (6.9%)	
**Secondary outcomes**												
Cardiac screening (ECG/Holter or Cardiologist assessment(% in last 6 months)	12,760 (55.1%)	24,571 (49.2%)	< .001	7,043 (51.6%)	15,566 (45.9%)	< .001	5,028 (59.0%)	7,884 (55.1%)	< .001	689 (71.6%)	1,121 (65.5%)	0.001
ECG or Holter	12,118 (52.4%)	23,341 (46.7%)	< .001	6,669 (48.8%)	14,774 (43.5%)	< .001	4,782 (56.1%)	7,489 (52.3%)	< .001	667 (69.3%)	1,078 (63.0%)	< .001
Cardiologist assessment	6,691 (28.9%)	11,881 (23.8%)	< .001	3,473 (25.4%)	7,089 (20.9%)	< .001	2,825 (33.1%)	4,189 (29.3%)	< .001	393 (40.9%)	603 (35.2%)	0.004
Duration of initial prescription												
Less than 30 days	9,673 (41.8%)	17,734 (35.5%)	< .001	5,094 (37.3%)	11,629 (34.3%)	< .001	4,152 (48.7%)	5,515 (38.5%)	< .001	427 (44.4%)	590 (34.5%)	< .001

*P* = P-value, ChEI = cholinesterase inhibitor.

ECG = electrocardiogram.

### Differences between prescribing patterns of male and female physicians

#### Primary outcome

Among all 9,254 physicians, female physicians were significantly more likely than male physicians to initiate ChEI therapy at a lower-than-recommended initial dose (6.1%% vs 4.1%) and the adjusted odds ratio (AOR) was 1.34 (95% confidence interval (CI) = 1.08–1.65) (Table 3). Among the 7,718 family medicine physicians, female physicians were significantly more likely than male physicians to initiate ChEI therapy at a lower-than-recommended initial dose (5.2% vs 3.7%) and the AOR was 1.31 (95% CI = 1.06–1.62) (Table 3). The trend was similar for dementia specialists (AOR = 3.68, 95% CI = 2.47–5.49), but not for non-dementia specialists (AOR = 1.27, 95% CI = 0.69–2.34).

**Table 3:** Association between physician sex and initial cholinesterase inhibitor dose prescribed.

Groups	Physician Sex	Unadjusted				Patient adjusted*			
		Lower-than-Recommended initial dose**		Higher-than- Recommended initial dose**		Lower-than-Recommended initial dose**		Higher-than- Recommended initial dose**	
		OR (95% CI)	P-value	OR (95% CI)	P-value	AOR (95% CI)	P-value	AOR (95% CI)	P-value
Overall	Male	1.00 (ref)		1.00 (ref)		1.00 (ref)		1.00 (ref)	
	Female	1.43 (1.17–1.74)	0.0004	0.90 (0.78–1.03)	0.1369	1.34 (1.08–1.65)	0.0068	0.89 (0.77–1.03)	0.1211
Family Medicine/General practice	Male	1.00 (ref)		1.00 (ref)		1.00 (ref)		1.00 (ref)	
	Female	1.33 (1.07–1.64)	0.0087	0.91 (0.79–1.04)	0.1775	1.31 (1.06–1.62)	0.0121	0.93 (0.80–1.07)	0.2847
Dementia Specialist (Geriatric Medicine, Psychiatry, Neurology)	Male	1.00 (ref)		1.00 (ref)		1.00 (ref)		1.00 (ref)	
	Female	3.81 (2.54–5.72)	< .0001	0.76 (0.53–1.08)	0.1198	3.68 (2.47–5.49)	< .0001	0.78 (0.55–1.10)	0.1572
* Geriatric Medicine*	Male	1.00 (ref)		1.00 (ref)		1.00 (ref)		1.00 (ref)	
	Female	2.84 (1.45–5.56)	0.0023	1.29 (0.83–2.00)	0.2587	2.76 (1.39–5.51)	0.0039	1.33 (0.85–2.09)	0.2087
* Psychiatry*	Male	1.00 (ref)		1.00 (ref)		1.00 (ref)		1.00 (ref)	
	Female	3.17 (1.58–6.37)	0.0012	0.58 (0.34–0.98)	0.0427	3.00 (1.48–6.08)	0.0023	0.60 (0.36–0.99)	0.0440
* Neurology*	Male	1.00 (ref)		1.00 (ref)		1.00 (ref)		1.00 (ref)	
	Female	3.27 (1.76–6.06)	0.0002	1.14 (0.57–2.28)	0.7168	3.25 (1.73–6.11)	0.0003	1.17 (0.58–2.35)	0.6593
Non Dementia Specialist	Male	1.00 (ref)		1.00 (ref)		1.00 (ref)		1.00 (ref)	
	Female	1.34 (0.74–2.45)	0.3355	1.05 (0.64–1.72)	0.8366	1.27 (0.69–2.34)	0.4358	1.02 (0.62–1.67)	0.9400

*Adjusted for patient age (continuous), sex, income quintile, rural residence, number of ADGs (continuous), acute myocardial infarction, asthma, angina, arrhythmia, cancer, diabetes, congestive heart failure, hypertension, stroke, liver disease, renal disease, mood/anxiety disorders, and number of concurrent medications used.

** Compared to recommended initial dose

ref: reference category.

Among the 739 dementia specialists, sex-specific differences were similar but higher than those found in family medicine alone (geriatricians (AOR = 2.76, 95% CI = 1.39–5.51), psychiatrists (AOR = 3.00, 95% CI = 1.48–6.08), and neurologists (AOR = 3.25, 95% CI = 1.73–6.11).

#### Secondary outcomes

Both female family physicians and specialists were more likely to have patients who had a cardiac screening prior to initiating a ChEI (55.1% vs. 49.2%, P-value<0.001). (Table 2).

Overall, female physicians were more likely to prescribe a shorter duration (30 days or less) of initial ChEI prescription (41.8% vs. 35.5%, P-value<0.001). Among the family medicine physicians, dementia and non-dementia specialists, female physicians were more likely to prescribe a shorter duration (30 days or less) of initial ChEI prescription (37.3% vs. 34.3%, P-value<0.001 for family medicine, 48.7% vs. 38.5%, P-value<0.001 for dementia specialist, and 44.4% vs. 34.5%, P-value<0.001 for non-dementia specialists).

## Discussion

Our study is among the first to demonstrate statistically significant and consistent differences in prescribing patterns between female and male physicians. This suggests that female prescribers have a tendency towards more careful and conservative prescribing practices when prescribing a widely used dementia drug therapy. This finding was consistent whether they were family medicine physicians, dementia specialist or other types of specialists. More specifically, our data indicate that female physicians were more likely than male physicians to follow the geriatric medicine maxim “*start low and go slow”* and prescribe a lower-than-recommended starting dose. We also demonstrated a similar pattern with other closely related measures. Specifically, patients prescribed ChEI by female physicians were more likely to have had cardiac screening (prior assessment by a cardiologist or ECG or Holter monitor) and to be dispensed a shorter duration of medication thereby facilitating monitoring for adverse events.

We are unaware of prior research that has studied sex and gender differences between male and female physicians in the prescribing process. Our findings concur with interdisciplinary research on sex and gender differences in risk perception and behaviour[32]. A meta-analysis examining 150 studies comparing risk-taking tendencies of men and women showed that almost all types of risk-taking were more frequent in men[33]. A study of college students asked men and women to report their perception and preferences related to risk-taking scenarios. Women were less likely to take risks and this was related to a perceived likelihood of a negative outcome[34]. This may be relevant to adverse drug events as they are perceived as a negative outcome. There is a suggestion from the financial sector research that women take fewer risks than men[35, 36]. A study from the investment industry found that women made smaller investments in risky assets than men[37]. Risk aversion, whether financial or in medical practice, may be a more frequent behaviour of women. Initiating a drug therapy at a low-dose, providing advanced cardiac screening for vulnerable patients, and providing the initial prescription for a shorter period of time may also be consistent with evidence suggesting that women are more cautious about preventing adverse drug events, and as such more careful and conservative prescribers.

Our results add to the growing evidence that there are differences in the processes of care received by patients managed by female physicians. This past year, Tsugawa et al[8] examined the outcomes of older hospitalized patients treated by internists. They found that those treated by female internists had lower mortality and readmission rate compared to similar patients cared for by male internists. Further, Wallis et al[9] found that patients treated by female surgeons had a decrease in 30-day mortality. Our study suggests these differences also exist for the process of drug prescribing. While we do not know if these differences in prescribing processes will also lead to improved outcomes, these findings will inform future research designed to determine if patients receiving lower-than-recommended initial doses also have better outcomes. We do know that adverse events are often related to the drug ordering and monitoring phase of the prescribing process[38]. It is important to understand these subtle prescribing differences so that we can apply this information to improve prescribing practices more generally.

Our choice of a lower-than-recommended initial dose as a measure of more careful and conservative prescribing is supported by our findings that geriatricians and other dementia specialists were more likely to initiate ChEI at low doses. Geriatricians are specialized in the care of older adult. They have had specialist training and education about how best to prescribe to vulnerable older adults. Thus, geriatricians’ prescribing practices for those with dementia could be regarded as the model to follow when prescribing for this population. For instance, geriatricians are taught to follow the *start low and go slow* philosophy when prescribing, particularly when the medication being prescribed is discretionary.

Prescribing a lower-than-recommended initial dose of a ChEI was not common in any specialty groups in our study. As expected, the vast majority (more than 80%) of prescribers were in family medicine. While dementia specialists are the group most likely to prescribe low-dose therapy, they account for only 1.3% of all prescribers. Given that relatively few older adults receive care from a geriatricians, it is important that geriatricians develop strategies to share their expertise in caring for the most vulnerable older adults more broadly[25].

### Limitations

First, we acknowledge that we focused on the use of ChEI therapy and recognize that this is only one type of drug therapy. Future research should explore this prescribing pattern for other widely used drug therapies where a more careful and conservative approach may be justified. Irrespective, ChEI therapy is prescribed to millions of older people globally, and as such subtle differences in prescribing processes could have a large impact at the population level. Second, there are many measures that could be used to evaluate more careful and conservative prescribing. We selected prescribing of lower-than-recommended doses of ChEI as our primary outcome measure. We recognize that the decision to initiate a therapy at this lower-than-recommended dose in an older person needs to be considered in the clinical context. For example, we recognize that starting low and being slow to titrate the dose could delay the modest benefit observed at the higher therapeutic doses. On the other hand, when prescribing a therapy of modest benefit such as a ChEI, it is important to do so in a way that minimizes the development of adverse events that may negatively impact quality of life and lead to the premature decision to discontinue the therapy before there is an opportunity for it to realize any benefit. Third, we used evidence of having a cardiac screening prior to initiating a ChEI as a measure of carefulness. While we do not know if the physician doing the prescribing ordered these investigations, we do know that the investigations were obtained prior to the prescription being dispensed. Finally, we identified providing an initial prescription of a shorter duration as a way to estimate closer monitoring. We acknowledge that receipt of a longer prescription does not preclude follow up. In some cases follow-up may happen in ways that are difficult to detect with administrative data such as by community pharmacists or by nurses or there could be physician follow up visits scheduled prior to the prescription renewal. Irrespective dispensing a shorter initial duration of the prescription is one way to facilitate the monitoring process.

Our study provides important new information about prescribing behaviours. Lessons learned from the more risk-averse, and more careful and conservative prescribing by female physicians, may help to inform better prescribing for all. When prescribing a therapy of modest benefit such as a ChEI, it is important to do so in a way that minimizes the development of adverse events that may impair quality of life. Given the frequency of prescribing for older adults, achieving this more careful and conservative approach to prescribing needs to be considered.

## Conclusions

Using a large cohort of older adults, our study found a consistent and statistically significant difference in prescribing patterns between female and male physicians. This finding suggests that female physicians may be more careful and conservative in their prescribing practices. This will inform future research to determine if patients receiving lower-than-recommended initial doses also have better outcomes.

## Supporting information

S1 File List of nine linkable administrative health care databases housed at the Institute for Clinical Evaluative Sciences (ICES). (DOCX)

S1 Table Dose categories for oral cholinesterase inhibitor therapy. (DOCX)
