# Supplementary material for: Comparison of prescribing practices for older adults treated by female versus male physicians: A retrospective cohort study
Source: PLoS One. 2018 Oct 22;13(10):e0205524. doi: 10.1371/journal.pone.0205524 (PMC6197851; doi:10.1371/journal.pone.0205524)
Supplement: S1 File — (DOCX) [file pone.0205524.s001.docx]

**S1 File. List of Nine linkable administrative health care databases housed at the Institute for Clinical Evaluative Sciences (ICES)**

1 The Registered Persons Database for demographic data

2 The Ontario Health Insurance Plan for physician billings

3 The Canadian Institute for Health Information Discharge Abstract Database for information on hospital admissions

4 The Ontario Drug Benefit Database for prescription drug benefit claims

5 The National Ambulatory Care Reporting System for emergency department visits

6 The ICES Physician database to describe physician specialty

7 The Ontario Cancer Registry for a record of close to 50 years of cancer history

8 The Office of the Registrar General for death records

9 Home Care Database
