# Supplementary material for: Comparison of prescribing practices for older adults treated by female versus male physicians: A retrospective cohort study
Source: PLoS One. 2018 Oct 22;13(10):e0205524. doi: 10.1371/journal.pone.0205524 (PMC6197851; doi:10.1371/journal.pone.0205524)
Supplement: S1 Table — (DOCX) [file pone.0205524.s002.docx]

**S1 Table. Dose Categories for Oral Cholinesterase Inhibitor Therapy**

| **Dose Category** | **Drug type** | | |
| --- | --- | --- | --- |
|  | **Donepezil** | **Galantamine** | **Rivastigmine** |
| Lower-than-recommended starting dose | <5 mg | <8 mg | < 3 mg |
| Recommended starting dose | 5 mg | 8 mg | 3 mg |
| Higher-than-recommended starting dose | 5.01-23 mg | 8.01 - 24 mg | 3.01 - 12 mg |

The lower than recommended starting dose for donepezil was half of the lowest manufactured dose, meaning that individuals in some cases may be required to “pill split” to obtain the desired dose. Mutually exclusive initial dose categories were created using the following steps: 1) based on data obtained in the Ontario Drug Benefit Database, a daily dose was estimated for each patient using strength of dispensed medication, quantity of medication dispensed, and number of days supplied; 2) for each cholinesterase inhibitor (donepezil, rivistigmine, galantamine), daily dose was categorized as one being at, below or above the recommended starting dose based on guidelines and our prior research. While the highest recommended dose in Canada for donepezil is 10 mg, in the United States a higher dose 23 mg tablet is available; as such we used 23 mg as the highest maximum dose, thereby allowing for off-label prescription. Doses that exceeded the highest recommended dose (i.e. 23 mg for donepezil, 12 mg for rivastigmine, 24 mg for galantamine) were excluded due to data accuracy concerns.
